# Supplementary material for: Impact of SARS-CoV-2 Alpha and Gamma Variants among Symptomatic Pregnant Women: A Two-Center Retrospective Cohort Study between France and Brazil
Source: J Clin Med. 2022 May 9;11(9):2663. doi: 10.3390/jcm11092663 (PMC9101133; doi:10.3390/jcm11092663)
Supplement: Supplementary file 1 [file jcm-11-02663-s001.zip › jcm-1625497-supplementary.pdf]

Journal of
Clinical Medicine

MDPI

Article

SUPPLEMENTARY MATERIAL

Impact of SARS-CoV-2 Alpha and Gamma Variants among Symptomatic Pregnant Women: A Two-Center Cohort Study between France and Brazil

Table S1. Baseline characteristics of COVID-19 symptomatic pregnant women during the "wild-type period" and "variant period" detailed by hospital.

Characteristic	Overall (1 February 2020–1 June 2021)	Wild-Type Period (1 February 2020–13 February 2021)			Variant period (14 February 2021–1 June 2021)		
		Overall	Béclère Hospital (France) (Wild-Type)	Sepaco Hospital (Brazil) (Wild-Type)	Overall	Béclère Hospital (France) (Alpha Variant)	Sepaco Hospital (Brazil) (Gamma Variant)
Total, N (%)	N=151/151	N=126/126	N=68/126	N=58/126	N=25/25	N=11/25	N=14/25
Mean age, years mean (IQR)	31.0 (28.0, 35.0)	31.0 (28.0, 34.0)	32.0 (29.0, 35.0)	29.5 (26.0, 34.0)	33.0 (30.0, 36.0)	31.0 (29.5, 34.0)	35.0 (32.2, 38.0)
Ethnic group, N (%)							
White	85 / 151 (56%)	70/126 (56%)	28/68 (41%)	42/58 (72%)	15/25 (60%)	3/11 (27%)	12/14(86%)
Black	27 / 151 (18%)	24/126 (19%)	24/68 (35%)	0/58 (0%)	3/25 (12%)	2/11 (18%)	1/14 (7.1%)
Maghrebian	18 / 151 (12%)	13/126 (10%)	13/68 (19%)	0/58 (0%)	5/25 (20%)	5/11 (45%)	0/14 (0%)
Hispanic	20 / 151 (13%)	18/126 (14%)	2/68 (2.9%)	16/58 (28%)	2/25 (8.0%)	1/11 (9.1%)	1/14 (7.1%)
Asian	1 / 151 (0.7%)	1/126 (0.8%)	1/68 (1.5%)	0/58 (0%)	0/25 (0%)	0/11 (0%)	0 14 (0%)
Pre pregnancy BMI, kg/m^2 mean (IQR)	26.2 (23.3, 30.3)	26. (23.0, 29.9)	25.0 (22.7, 29.0)	27.1 (24.7, 30.3)	29.0 (26.0, 35.0)	26.0 (23.5, 27.5)	31.2 (29.3, 35.2)
Parity, N (%)							
Nulliparous	73/151 (48.3%)	59/126(46.9%)	32/68 (47.1%)	27/58 (46.6%)	14/25 (56.0%)	5/11 (45.5%)	9/14 (64.3%)
Multiparous	78/151 (51.7%)	67/126(53.1%)	36/68 (52.9%)	31/58 (53.4%)	11/25 (44.0%)	6/11 (54.5%)	5/14 (35.7%)
Current smoking, N(%)	16/151 (11%)	13/126 (10%)	7/68 (10%)	6/58 (10%)	3/25 (12%)	2/11 (18%)	1/14 (7.1%)
Pre-existing medical conditions, N(%)	38/151 (25%)	29/126 (23%)	15/68 (22%)	14/58 (24%)	9/25 (36%)	4/11 (36%)	5/14 (36%)
Chronic hypertension	14/151 (9.3%)	13/126 (10%)	3/68 (4.4%)	10/58 (17%)	1/25 (4.0%)	0/11 (0%)	1/14 (7.1%)
Diabetes mellitus	1/151 (0.7%)	0/126 (0%)	0/68 (0%)	0/58 (0%)	1/25 (4.0%)	0/11 (0%)	1/14 (7.1%)
Pulmonary disease (including asthma)	11/151 (7.3%)	8/126 (6.3%)	4/68 (5.9%)	4/58 (6.9%)	3/25 (12%)	2/11 (18%)	1/14 (7.1%)
Others	12/151 (7.9%)	8/126 (6.3%)	8/68 (11.8%)	0/68 (0%)	4/25 (16%)	2/11 (18.2%)	2/14 (14.3%)
Multiple pregnancies, N (%)	8/151 (5.3%)	7/126 (5.6%)	5/68 (7.4%)	2/58 (3.4%)	1/25 (4.0%)	1/ 11 (9.1%)	0/ 14 (0%)
COVID-19-related information							
Gestational age at the time of positive RT-PCR, weeks mean (IQR)	32 (26, 35)	31 (26, 35)	32 (26, 35)	31 (26, 36)	34 (32, 36)	34 (33, 35)	33 (32, 36)
Hospital admission for COVID-19-related illness, N (%)	57/151 (38%)	43/126 (34%)	21/68 (31%)	22/58 (38%)	14/25 (56%)	5/11 (45%)	9/14 (64%)

BMI: body mass index; IQR: interquartile range.

Table S2. Disease severity, obstetrical and neonatal outcomes among symptomatic pregnant women during the "wild-type period" and "variant period" (Alpha) in Béclère Hospital (Clamart, France).

Outcomes in Clamart (France)	Wild-Type Period 1 February 2020–13 February 2021 Women N=68 Neonates N=71	Alpha Period 14 February 2021–1 June 2021 Women N=11 Neonates N=10	Univariate Analysis		
			OR	95% CI	*p*-Value
Severe to critical infection according to WHO	17/68 (25.0%)	6/11 (54.6%)	3.25	0.70, 15.6	0.13
Composite adverse obstetric outcome	27/68 (39.7%)	7/11 (63.6%)	2.45	0.63, 10.8	0.2
Composite adverse neonatal outcome	27/71 (38.0%)	3/10 (30.0%)	0.50	0.09, 2.17	0.4

WHO World Health Organization; OR: odds ratio; CI: confidence interval.

Table S3. Disease severity, obstetrical and neonatal outcomes among symptomatic pregnant women during the "wild-type period" and "variant period" (Gamma) in Sepaco Hospital (Sao Paulo, Brazil)

Outcomes in Sao Paulo (Brazil)	Wildtype Period 1 February 2020–13 February 2021 Women N=58 Neonates N=60	Gamma Period 14 February 2021–1 June 2021 Women N=14 Neonates N=14	Univariate Analysis		
			OR	95% CI	*p*-Value
Severe to critical infection according to WHO	5/58 (8.6%)	7/14 (50%)	11.8	2.46, 72.3	0.003
Composite adverse obstetric outcome	24/58 (41%)	8/14 (57%)	1.06	0.23, 4.87	>0.9
Composite adverse neonatal outcome	15/60 (25%)	7/14 (50%)	2.69	0.77, 9.42	0.12

WHO World Health Organization; OR: odds ratio; CI: confidence interval.
